# ETV2 and VEZF1 interaction and regulation of the hematoendothelial lineage during embryogenesis

**DOI:** 10.3389/fcell.2023.1109648

**Published:** 2023-02-27

**Authors:** Satyabrata Das, Vinayak Gupta, Johannes Bjorge, Xiaozhong Shi, Wuming Gong, Mary G. Garry, Daniel J. Garry

**Affiliations:** ^1^ Department of Medicine, Cardiovascular Division, Lillehei Heart Institute, University of Minnesota, Minneapolis, MN, United States; ^2^ Department of Physiology, Basic Medical College, Nanchang University, Nanchang, JX, China; ^3^ Stem Cell Institute, University of Minnesota, Minneapolis, MN, United States; ^4^ Paul and Sheila Wellstone Muscular Dystrophy Center, University of Minnesota, Minneapolis, MN, United States

**Keywords:** ETV2/ER71, VEZF1, transcriptional (regulation), hematopoietic cell development, stem cell, endothelial development

## Abstract

Ets variant 2 (Etv2), a member of the Ets factor family, has an essential role in the formation of endothelial and hematopoietic cell lineages during embryonic development. The functional role of ETS transcription factors is, in part, dependent on the interacting proteins. There are relatively few studies exploring the coordinated interplay between ETV2 and its interacting proteins that regulate mesodermal lineage determination. In order to identify novel ETV2 interacting partners, a yeast two-hybrid analysis was performed and the C2H2 zinc finger transcription factor VEZF1 (vascular endothelial zinc finger 1) was identified as a binding factor, which was specifically expressed within the endothelium during vascular development. To confirm this interaction, co-immunoprecipitation and GST pull down assays demonstrated the direct interaction between ETV2 and VEZF1. During embryoid body differentiation, *Etv2* achieved its peak expression at day 3.0 followed by rapid downregulation, on the other hand *Vezf1* expression increased through day 6 of EB differentiation. We have previously shown that ETV2 potently activated *Flt1* gene transcription. Using a *Flt1* promoter-luciferase reporter assay, we demonstrated that VEZF1 co-activated the *Flt1* promoter. Electrophoretic mobility shift assay and Chromatin immunoprecipitation established VEZF1 binding to the *Flt1* promoter. *Vezf1* knockout embryonic stem cells had downregulation of hematoendothelial marker genes when undergoing embryoid body mediated mesodermal differentiation whereas overexpression of VEZF1 induced the expression of hematoendothelial genes during differentiation. These current studies provide insight into the co-regulation of the hemato-endothelial lineage development *via* a co-operative interaction between ETV2 and VEZF1.

## Introduction

The pioneer regulator, ETS variant 2 (*Etv2*, also known as *Er71/Etsrp71*), a member of the ETS (E26 transformation-specific) transcription factor family, has critical and essential roles for hematoendothelial lineages during embryonic development. One role for ETV2 is the transcriptional activation of several genes critical for both hematopoiesis and vascular development including *Flt1*, *Lmo2*, *Scl*, *Tie2*, *Sox7* and others (De Val et al., 2008; Kataoka et al., 2011; Rasmussen et al., 2011; Koyano-Nakagawa et al., 2012; Lammerts van Bueren and Black, 2012; Liu et al., 2012; Wareing et al., 2012; Abedin et al., 2014; Behrens et al., 2014; Shi et al., 2014; Koyano-Nakagawa et al., 2015). During development, *Etv2* has a narrow window of expression from E7.0-E9.5 after which it is downregulated and relatively extinguished by E11.5. Biallelic disruption of the *Etv2* gene results in the absence of vascular and hematopoietic lineages in both the yolk sac and the embryo resulting in lethality of the null mouse embryo by E9.5 (Ferdous et al., 2009; Koyano-Nakagawa et al., 2012). Recent studies have shown that ETV2 functions as a pioneer factor supporting the notion that it resides at the top of the hierarchy for the regulators that govern the development of the hematoendothelial lineages (Gong et al., 2022).

Studies in zebrafish and *Xenopus* also supported the notion that ETV2 had an essential role during cardiovascular development (Sumanas and Lin, 2006; Neuhaus et al., 2010). ETS factors including ETV2 harbor an evolutionarily conserved winged helix-turn-helix domain (85-amino acids) that binds to a core GGA (A/T) motif. Although previous studies have shown that the role of ETV2 is partially mediated through the direct interaction with factors in a context dependent fashion (Sharrocks, 2001; Hollenhorst et al., 2011), very few studies have probed the role of ETV2 interacting proteins (Knebel et al., 2006; De Val et al., 2008; Kim et al., 2014; Shi et al., 2014). These studies defined GATA2, FOXC1/2, JMJD2A/2D, BRG1, OVOL2 and others which amplified and/or promoted the functional role for ETV2. Therefore, further studies are needed to define ETV2 interacting factors.

VEZF1 is a putative zinc finger transcription factor that harbors 6 (Cys2-His2)-type (Kruppel-like) zinc finger motifs, a poly glutamine-domain and a proline-rich region and plays an essential role during angiogenesis (Koyano-Nakagawa et al., 1994; Xiong et al., 1999; Bruderer et al., 2013). *Vezf1* expression was initially detected in the mesodermal components of the extraembryonic as well as the embryo proper. Later, the VEZF1 expression of VEZF1 was localized to endothelial cells arising during vasculogenesis and angiogenesis (Xiong et al., 1999). *Vezf1* mRNA expression was detected from E7.25 to E11.5 in mice, and co-expressed with other endothelial cell-specific factors (Xiong et al., 1999). Despite its expression during early stages of endothelial differentiation, VEZF1 does not transactivate the *Flk1* promoter (an early angioblast protein). On the other hand, it strongly transactivates the endothelin-1 promoter (an endothelial cell restricted promoter) (Aitsebaomo et al., 2001). Apart from these studies, VEZF1 regulates distinct stages of angiogenesis by targeting downstream genes including metallothionein 1 (MT1) and stathmin/OP18 (Miyashita et al., 2004; Miyashita and Sato, 2005). Besides, *Vezf1* null ES cell-derived embryoid bodies have perturbed vascular networks and structural defects resulting in sprouting and blood vessel malformations, but with no impairment in endothelial differentiation (Zou et al., 2010). Moreover, deletion of *Vezf1* during mouse embryogenesis resulted in lethality due to vascular structural defects demonstrating that VEZF1 is an essential regulator of vasculogenesis (Kuhnert et al., 2005). Identification of VEZF1 endothelial specific targets will enhance our understanding of the role of VEZF1 in vascular development.

In the current study, we discovered the interaction between ETV2 and VEZF1. This interaction plays an important role in the transcriptional regulation of the *Flt1* gene. Direct binding of VEZF1 to this previously known downstream target of ETV2 showed the co-operative role played by the two transcription factors in co-regulating embryoid body mediated hematoendothelial lineage development. In addition, the absence and overexpression of VEZF1 in embryoid bodies resulted in down- and up-regulation of hematoendothelial marker genes, respectively during early mesodermal lineage differentiation.

## Materials and methods

### Generation of doxycline inducible mouse embryonic stem cells, and embryoid body differentiation assays

Doxycycline-inducible HA-Etv2 overexpressing mouse embryonic stem cells (iHA-Etv2-mES) were generated in our laboratory and have been described previously (Koyano-Nakagawa et al., 2012). Similarly, to generate doxycycline-inducible *Vezf1* mouse embryonic stem cells, we subcloned *Vezf1* cDNA from pCMV-Vezf1-myc-flag (Origene) plasmid into the p2Lox plasmid. The authenticity of p2Lox-*Vezf1-Myc-Flag* plasmid was confirmed by sequencing using p2lox-FP:5′-CTTTCGACCTGCATCCATCT-3′ and p2lox-RP: 5′-TGG​TTT​GTC​CAA​ACT​CAT​CAA-3’.

Mouse ES cells were cultured and differentiated into embryoid bodies as previously described (Iacovino et al., 2014). EBs were prepared using the hanging drop technique and the appropriate mES cell lines, and then cultured in suspension beginning on day 2. For induction of *HA-Etv2* or *Vezf1-Flag* separately or together, the respective mES cells were treated with 0.5 μg/mL doxycycline. Western blot analysis and qPCR were performed (as described below) to verify the over-expression of ETV2 and VEZF1.

### Generation of *Vezf1* knockout mouse embryonic stem cells

To generate the *Vezf1* knockout mES, the exon two of Vezf1 was deleted in V6.5 ES cells using the CRISPR/Cas9 system as previously described (Ran et al., 2013). The guide RNAs (gRNAs) targeting the 5′ and 3’ ends of *Vezf1* exon *2* (Table 1) were cloned into the Cas9 expressing plasmid pSpCas9(BB)-2A-Puro (PX459) (Addgene, # 48139). V6.5 mES cells were transfected with both gRNA expression plasmids and 24 h post-transfection, cells were treated with 2 μg/mL puromycin for 36 h followed by seeding on mouse embryonic fibroblast (MEF) cells. Single colonies were picked after 5–7 days for expansion and genotyped using PCR. The wild-type (wt) cells yielded a 1754 bp product, whereas a homozygous deletion of the exon two yielded a 677 bp product. The PCR amplified products were cloned into the pCR2.1 TOPO plasmid using TOPO^®^ TA Cloning^®^ Kit (Invitrogen), and the biallelic deletion was verified using the Sanger sequencing technique.

**TABLE 1:** gRNAs used for generating Vezf1 ko ES cells.

mmVezf1 intron 1 gOligo4F	CAC​CGA​GAA​CAG​TTC​ATA​GGC​TCC
mmVezf1 intron 1 gOligo 4R	AAA​CGG​AGC​CTA​TGA​ACT​GTT​CTC
mmVezf1 intron 2 gOligo4F	CAC​CGC​TTT​ATA​TCC​GGT​TCT​CCA​A
mmVezf1 intron 2 gOligo4R	AAA​CTT​GGA​GAA​CCG​GAT​ATA​AAG​C

### Total RNA extraction and quantitative real-time PCR

Total RNA was extracted from mouse ES cells or embryoid bodies (harvested on different days of differentiation) using TRIzol Reagent (Invitrogen) and purified with the RNeasy Mini Kit (Qiagen). Following DNAse treatment, reverse transcription was performed with 500 ng of RNA using High Capacity cDNA Reverse Transcription Kit (Applied Biosystems). Quantitative Real-Time PCR analyses were performed using an ABI PRISM 7,900 sequence detection system and gene specific TaqMan probes. Probes used included: VIClabeled Gapdh: 4352339E, FAM labeled Etv2: mm01176581_g1, Vezf1: Mm00497288_m1, Flk1: mm00440099_m1, Pecam: mm01246167_m1, Tie2: mm01256892_m1, VEGFa: mm00437304_m1, Scl: mm01187033_m1, Cdh5: mm00486938_m1, Gata1: mm00484678_m1, Gata2: mm00492300_m1, c-kit: mm00445212_m1, Lmo2: mm00493153_m1, Fli1: mm00484410_m1, PU1: mm0048140_m1, CD41: mm00439741_m1, Runx1: mm00486762_m1, CD34: mm00519283_m1, and Endoglin: mm00468262_g1. Each transcript level was normalized to Gapdh expression.

In studies using inducible overexpression *Vezf1-ES* cells, the primer pair mVezf1-qRT-F1: 5′-ACA​TTA​GCT​GCC​CCT​CTC​AA-3′ and mVezf1-3′UTR-QRT-R1: 5′-ATT​TAC​TTT​AAC​CCA​ATT​TTG ACTTT-3′ were used to detect the endogenous Vezf1 gene specifically (not the overexpressed Vezf1). On the other hand, the transcript level of over-expressed *Vezf1* (not the endogenous *Vezf1* transcript) was assessed using mVezf1-qRT-F1 and myc-mVezf1-qRT-R1: 5′-TTGCTG CCAGATCCTCTTCT-3’. The Vezf1 expression was normalized to Gapdh expression using mGapdh-RT-FP: 5′-TGT​GTC​CGT​CGT​GGA​TCT​GA-3′ and mGapdh-RT-RP: 5′-CCT​GCT​TCA​CCA​CCT​TCT​TGA-3’.

### Co-immunoprecipitation and immunoblotting assays

To probe the interaction between HA-Etv2 and endogenous VEZF1, EBs (derived from dox-inducible HA-Etv2 mouse ES cells) were treated with doxycycline (0.5 μg/mL) from days 2–3 and then lysed using Co-IP lysis buffer (20 mM HEPES-KOH pH 7.9, 150 mM NaCl, 0.5% NP40, 1 mM EDTA, 10% Glycerol) supplemented with 1X protease inhibitor cocktail (Sigma), 1 mM PMSF (Sigma), 1 mM sodium orthovanadate, 10 mM sodium fluoride on day 4 for immunoprecipitation. Following centrifugation at 13.2 K rpm at 4°C, protein concentration was estimated in cell lysates using the BCA protein assay kit (Pierce). Immunoprecipitation and immunoblotting reactions were performed using Mouse IgG TrueBlot^®^ Protocol (Rockland, # 88–7,788–31). Immunoprecipitation reactions were performed using 1 mg of cell lysates and 2–5 μg of antibodies [Mouse anti-HA (12CA5) antibody from Roche, Mouse IgG TrueBlot antibody and ETV2 antibody (abcam #ab181847)] for the co-immunoprecipitation assays. Briefly, immunoprecipitated and input samples were separated by SDS-PAGE and transferred to activated PVDF membrane (Bio-Rad). After blocking with 5% non-fat milk for 1 h at room temperature, the membranes were incubated with specific primary antibody rat Anti-HA (Roche) at 1:1,000 dilution or VEZF1 antibody (Abcam, #ab50970) at 1:150 dilution, overnight at 4°C. After washing with 1X TBST, the membrane was incubated with rat or mouse HRP-conjugated secondary antibody (Santa Cruz Biotechnologies, United States of America, sc-2006 or #sc-2005) at 1:2,000 dilution) for 1 h. The detection was carried out using chemiluminescence kit (Pierce) and the signal was captured using the GelDoc Chemiluminescence Detection system (Bio-Rad).

### *In vitro* transcription, translation and pull down assays

For the pull down assays, GST-tagged constructs harboring full length ETV2 (1–335), N-terminal ETV2 (1–226) and C-terminal ETV2 (227–335) were generated using pGEX-4T1 vector as the backbone in our laboratory and have been previously described. The GST fusion proteins were induced using 0.5 mM isopropyl-D-thiogalactopyranoside (IPTG), at an optical density of 0.6, for 3 h at 25°C in BL21 cells. Cells were lysed using the B-PER protein extraction reagent (Pierce) containing 1X protease inhibitor cocktail, 1 mM PMSF, DNAse and lysozyme according to manufacturer’s instructions. GST fusion proteins were purified using Glutathione Sepharose 4B beads (GE Healthcare). The expression of GST fusion proteins in the input samples was confirmed by western blotting using anti-GST antibody (Bethyl laboratories, #A190-122A) and anti-rabbit secondary antibody (Santa Cruz Biotechnologies, United States of America, #sc-2004). Mouse VEZF1 was *in vitro* translated using TNT^®^ T7 Quick Coupled Transcription/Translation Systems (Promega) and pCMV-VEZF1-Flag-Myc (Origene) plasmid. Following the binding of GST fusion proteins to Glutathione Sepharose 4B beads, the beads were washed three times with cold 1X PBS and blocked overnight with BSA (2 mg/mL) to prevent the non-specific binding of VEZF1 with the beads. Beads bound with GST fusion proteins were incubated with 6 μL of *in vitro* translated VEZF1 protein for 2 h at 4°C with end-over-end rotation. After extensive washes with cold 1X PBS, proteins bound to the beads were eluted by the addition of elution buffer (10 mM glutathione, pH 8.0). Input and the eluted proteins were analyzed by western blotting using standard procedures. The myc and flag tagged VEZF1 was detected using mouse ant-Flag antibody (Sigma, #F1804 at 1:2,500) and anti-mouse secondary antibody (Santa Cruz Biotechnologies, United States of America, #sc-2005, at 1:2,000).

### Cell culture, co-transfection and luciferase assays

NIH3T3 and HEK-293T cells were maintained in DMEM containing high glucose, L-glutamine and sodium pyruvate (HyClone) supplemented with 10% (v/v) fetal bovine serum (HyClone), penicillin G and streptomycin sulfate (Invitrogen) in a humidified incubator at 37°C with 5% CO_2_. Luciferase assays were performed using a pGL3T-*Flt1* promoter reporter construct that was generated in our laboratory and has been previously described. For co-transfection and luciferase assays, NIH3T3 cells were grown in 24-well plates (70% confluence) and transfections were performed in triplicates with 250 ng/well of the pGL3T-*Flt1* promoter reporter construct and different doses (50, 100, 200, 400 ng) of *pcDNA-HA-Etv2* or *pCMV-Vezf1-Flag-Myc* (OriGene) expression plasmid using Lipofectamine 3,000 (Invitrogen) according to manufacturer’s instructions. Separately, the pGL3T-*Flt1* construct was co-transfected into NIH3T3 cells with single dose of Etv2/Vezf1 and different doses of Vezf1/Etv2 expression plasmids. In all these co-transfection experiments, the total amount of DNA was made equal using pcDNA3 as balancing plasmids in different transfection mixtures. Cells were lysed and luciferase assays were performed using the Dual-Luciferase assay (Promega) following the directions outlined in the user manual.

### Electrophoretic mobility shift assays (EMSAs) and chromatin immunoprecipitation (ChIP) assays

Gel shift assays were performed as described previously (Sasi et al., 2014; Sonawane et al., 2014). Nuclear protein extracts from HEK-293T cells were prepared using the NE-PER^®^ Nuclear and Cytoplasmic Extraction Reagents kit (Pierce) and stored in aliquots at −80°C until use. Wild type and mutant oligonucleotides (Table 2) were synthesized at IDT. The single stranded oligos were labeled at the 3′-end by using Biotin-11-UTP (Thermo Fisher Scientific) and terminal transferase (NEB) following the manufacturer’s protocol. Following the biotinylation, the terminal transferase was heat-inactivated at 70°C and oligos were purified using chloroform:isoamyl alcohol (25:24) (Sigma). Equal amounts of each oligomer (labeled or unlabled) and its complementary strand (labeled or unlabled) were annealed. Electrophoretic Mobility Shift assays were performed by using the LightShift Chemiluminescent EMSA kit (Pierce) and the signal was captured using the GelDoc Chemiluminescence Detection system (Bio-Rad). In some EMSA experiments, the nuclear extracts were incubated with 2X-, 4X-, and 8X-fold molar excess of the unlabeled oligomers having either wild type or mutated sequences (Table 1) in the binding buffer prior to the addition of biotin-labeled probes. Supershift assays were carried out using anti-VEZF1 antibodies (#sc-365560x, Santa Cruz Biotechnologies, United States).

**TABLE 2:** Wild-type and mutant oligonucleotides used for gel shift assays.

Name of the oligonucleotide	Sequence (5′ to 3′)
FLT1-VEZF1-WT-Oligo3-FP	5′- CCC​TCG​CCGGCCCCCGCC​CCT​CCG -3′
FLT1-VEZF1-WT –Oligo3-RP	5′-CGG​AGG​GGC​GGG​GGC​CGG​CGA​GGG -3′
FLT1-VEZF1-WT-Oligo4-FP	5′-GCG​GAT​GAG​GGGTGGGGGACC​CCT​TGA​CGT-3′
FLT1-VEZF1-WT –Oligo4-RP	5′-ACG​TCA​AGG​GGT​CCC​CCA​CCC​CTC​ATC​CGC -3′
FLT1-VEZF1-SDM7-FP	5′-GCG​GAT​GAG​ATGTGATGGACA​TCT​TGA​CGT-3′
FLT1-VEZF1-SDM7-RP	5′-ACG​TCA​AGA​TGT​CCA​TCA​CAT​CTC​ATC​CGC-3′
FLT1-VEZF1-SDM8-FP	5′-GCG​GAT​GAG​GGG​TGG​GGGACATCTTGA​CGT-3′
FLT1-VEZF1-SDM8-RP	5′-ACG​TCA​AGA​TGT​CCC​CCA​CCC​CTC​ATC​CGC-3′

To perform the ChIP assay, embryoid bodies (EBs) were prepared from doxycylcine-inducible *HA-Etv2* mouse ES cells, harvested at day 4.0 and dissociated with trypsin to obtain a single cell suspension. ChIP assays were performed as previously described (Sasi et al., 2014; Sonawane et al., 2014). Overnight immunoprecipiation reactions were performed using 5 µg of ChIP-grade antibodies against VEZF1 (#sc-365560x Santa Cruz Biotechnology) and mouse non-immune IgG as the negative control (Sigma). The immune complexes were captured using Rec protein G-Agarose beads (Invitrogen). The immune complexes were reverse cross-linked, treated with RNAse A/proteinase K, purified by PCR product purification kit (Promega) and stored at −20°C until used. End-point PCR as well as quantitative real-time PCR were carried out using immunoprecipitated chromatin as the template and the following m*Flt1*-specific primers: (forward: 5′-CTG​TCC​GGC​GAC​CCG-3′ and reverse 5′-TCC​CCA​GCC​CAC​TTC​CTA​C-3′). To demonstrate the specificity of the ChIP assay, Gapdh specific primer pair (forward: 5′-CCC​TTT​TCT​GCC​TTC​CTA​CC -3′ and reverse 5′-TGC​TGA​AGT​GCT​CCC​TAC​CT-3′) were designed and used to amplify a 176 bp promoter fragment without the VEZF1 binding domain. qPCR was performed using SsoAdvanced universal SYBR Green Supermix (Bio-Rad) and the fold enrichment of the *Flt1* promoter domain in the immunoprecipitates was quantified.

### Data representation and statistical analysis

All co-transfection experiments were performed at least three times and results were expressed as mean ± S.E. of triplicates from representative experiments. Statistical analyses were carried out using Student’s *t*-test or one-way ANOVA with Bonferroni’s multiple comparisons post-test, as appropriate, by using GraphPad Prism 5.

## Results

### ETV2 and VEZF1 expression during EB differentiation

**FIGURE 1:**
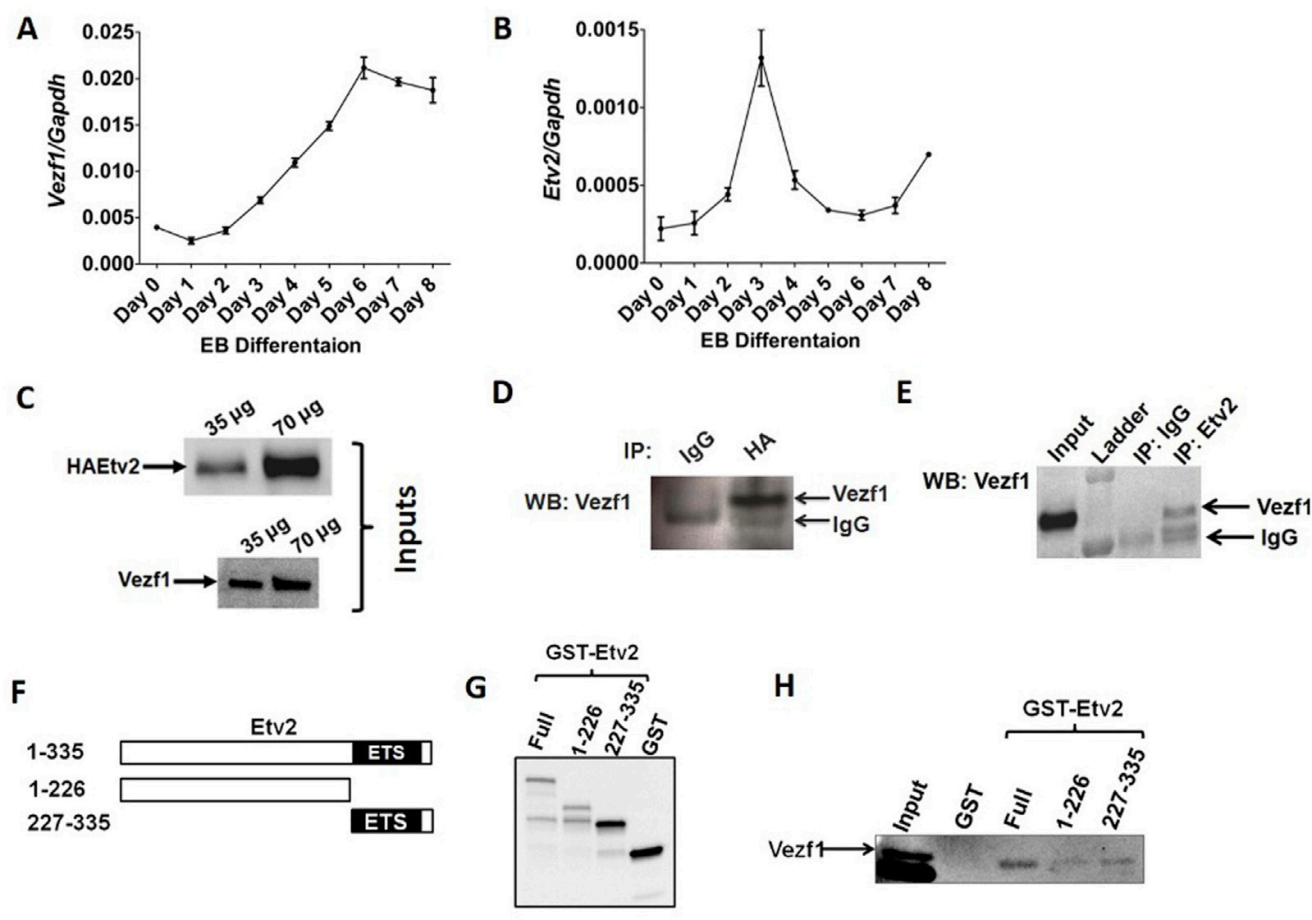
Expression pattern and interaction of *Vezf1* and *Etv2* during embryoid bodies differentiation. Embryoid bodies derived from iHA-Etv2-mES cells were harvested at different days of differentiation. Quantitative RT-PCR expression analysis of V*ezf1* **(A)** and *Etv2* **(B)** *.* The transcript level of *Vezf1* and *Etv2* were normalized to *Gapdh* expression. **(C–D)** Endogenous interaction of VEZF1 with HA-ETV2 by Co-immunoprecipitation assay. Expression of VEZF1 and ETV2 was confirmed in D4 EBs input lysates by Western blotting (panel C). VEZF1 was specifically detected in the western blot analysis of the pulled-down material using an anti-HA antibody to pull down HA-ETV2 from D4 EB protein lysates. Pre-immune IgG was used as a negative control (panel D). **(E)** Western blot showing the presence of VEZF1 using an ETV2 antibody for immunoprecipitation from D4 EBs. **(F–H)** Schematic illustration of ETV2 and its deletion constructs **(F)**. Expression of GST- Full length ETV2 and GST-tagged deletion constructs having 1–226 and 227–335 amino acids was confirmed in input samples by using anti-GST antibody in Western blot **(G)**. Direct interaction of VEZF1 with Full length ETV2 and GST-tagged deletion constructs was observed when *in vitro* translated VEZF1 was pulled down with GST-tagged full length deletion constructs of ETV2, but not with GST alone (negative control) **(H)**.

To evaluate the relationship between *Etv2* and *Vezf1* transcript expression patterns were examined during EB differentiation (derived from iHA-ETV2-mES cells), beginning on day 0 until day 8 (Figures 1A, B). As previously reported (Lee et al., 2008), *Etv2* expression showed a transient expression pattern peaking at day 3.0 and subsequently downregulated to baseline levels (Figure 1B). On the other hand, *Vezf1* expression remained similar from Day 0 to Day 2 and gradually increased from day 3 through day 6 EB differentiation (Figure 1A). Thus, *Etv2* and *Vezf1* had overlapping expression during EB mediated differentiation of ES cells on day 2–4.

### VEZf1 physically interacts with the pioneer factor, ETV2

Initially, VEZF1 was selected from a yeast two hybrid screen as a candidate to investigate its interaction with ETV2 because of its expression in the nucleus and its role in endothelial cell biology (Bruderer et al., 2013). Co-expression of *Etv2* and *Vezf1* in mES cells on days 2–4, further supported the hypothesis that ETV2 and VEZF1 could function *via* a protein–protein interaction mechanism. We performed co-immunoprecipitation (Co-IP) assay in differentiated EBs derived from doxycycline inducible iHA-Etv2-mES cell line which over expressed HA-tagged ETV2. Following Dox (0.5 μg/mL) treatment on day 2–3, the induction of *Etv2* transcript and protein was confirmed by qPCR and western blot techniques (Supplementary Figures S1A–C). As positive controls, input samples showed HA-tagged ETV2 and VEZF1 in cell lysates (Figure 1C). Endogenous VEZF1 was successfully immunoprecipitated using an anti-HA antibody while the negative control, non-immune mouse IgG did not precipitate VEZF1 (WB: anti-VEZF1, Figure 1D), thus confirming the specificity of the interaction. Furthermore, we pulled down VEZF1 from D4 EB lysates using an antibody against the endogenous ETV2 (Figure 1E). Together these studies establish the interaction between endogenous ETV2 and VEZF1.

To confirm the endogenous interaction between ETV2 and VEZF1, we performed an *in vitro* pulldown assay using GST-tagged ETV2 constructs as bait proteins and *in vitro* translated myc-flag-tagged VEZF1 as prey. Expression and purification of GST tagged full length (1–335 amino acids) or deletional N-terminal ETV2 (1–226 amino acids) and C-terminal ETV2 (227–335 amino acids) were verified by western blot (Figures 1F, G). *In vitro* translated myc-flag-tagged VEZF1 was pulled down with the full length, N-termina, and C-terminal ETV2 constructs, but not with the GST alone as control bait (Figure 1H). Collectively, these results supported the conclusion that ETV2 can directly bind with VEZF1 through the N- and C-terminal domains.

### Coordinated VEZF1 and ETV2 transactivation of the *Flt1* gene

**FIGURE 2:**
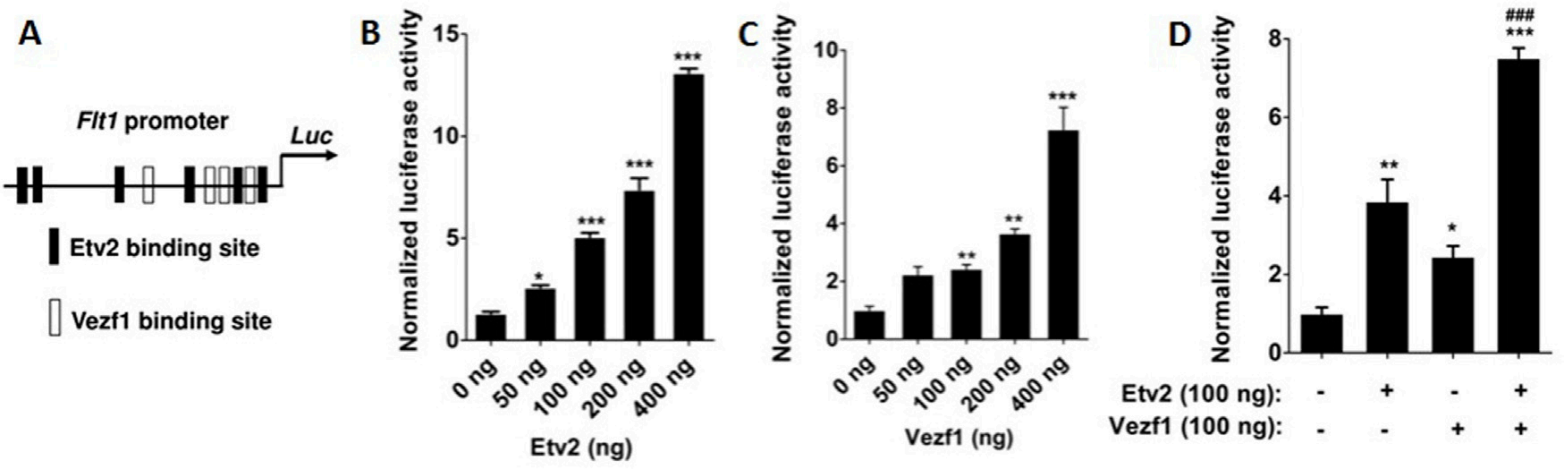
Transactivation of *Flt1* promoter by ETV2 and VEZF1. **(A)** Schematic presentation of VEZF1 and ETV2-binding sites in *Flt1* proximal promoter. **(B–C)** Modulation of *Flt1* promoter activity by ectopic expression of ETV2 and VEZF1. NIH3T3 cells were transiently transfected with increasing doses of VEZF1 **(B)** and ETV2 **(C)** expression plasmid along with *Flt1* promoter luciferase reporter plasmid. *Flt1* promoter activities were reported as fold over the basal promoter activity. **(D)** Co-transfection with VEZF1 and ETV2 expression plasmids shows an additive effect on the *Flt1* promoter activity. Data shown in panels B–D are shown as mean ± S.E. of triplicate values. Statistical significance was determined by one-way ANOVA with Bonferroni’s multiple comparisons post-test. *p < 0.05, **p < 0.01, ***p < 0.001, and ****p < 0.0001 with respect to basal promoter reporter activity.

The 0.7 kb *Flt1* promoter is essential for endothelial cell formation (Morishita et al., 1995; Jin et al., 2009) and we have previously shown that ETV2 potently transactivated the *Flt1* promoter (Koyano-Nakagawa et al., 2015). Therefore, in order to probe the functional consequences of ETV2 and VEZF1 interaction, we analyzed the *Flt1* promoter for ETV2/VEZF1 binding motifs and identified four putative VEZF1 binding motifs (Figure 2A). Hence, we tested the effect of ETV2/VEZF1 over-expression on *Flt1* promoter activity. Indeed, ETV2 and VEZF1 caused a dose-dependent increase in *Flt1* promoter reported luciferase activity by ∼12.0 (*p* < 0.001) and ∼7.3-fold (*p* < 0.001), respectively (Figures 2B, C). Both ETV2 and VEZF1 were able to enhance the activation of the *Flt1* promoter activity in a dose-dependent manner (Supplementary Figures S2A–B) when either of them were kept at a constant level. Next, we examined whether transcription of the *Flt1* gene was regulated by these transcription factors in an additive or synergistic manner. Accordingly, NIH3T3 cells were co-transfected with *Flt1* promoter plasmid and different concentrations of ETV2 and VEZF1. While ETV2 and VEZF1 enhanced the *Flt1* promoter activity by ∼ 4-fold (*p* < 0.01) and ∼ 2.5-fold (*p* < 0.05), respectively, the combination of both caused a more pronounced increase (∼7.5-fold, *p* < 0.001) in promoter activity (Figure 2D), suggesting an additive effect of ETV2 and VEZF1 in *Flt1* gene regulation.

### *In vitro* and endogenous interactions of VEZF1 with the *Flt1* promoter

**FIGURE 3:**
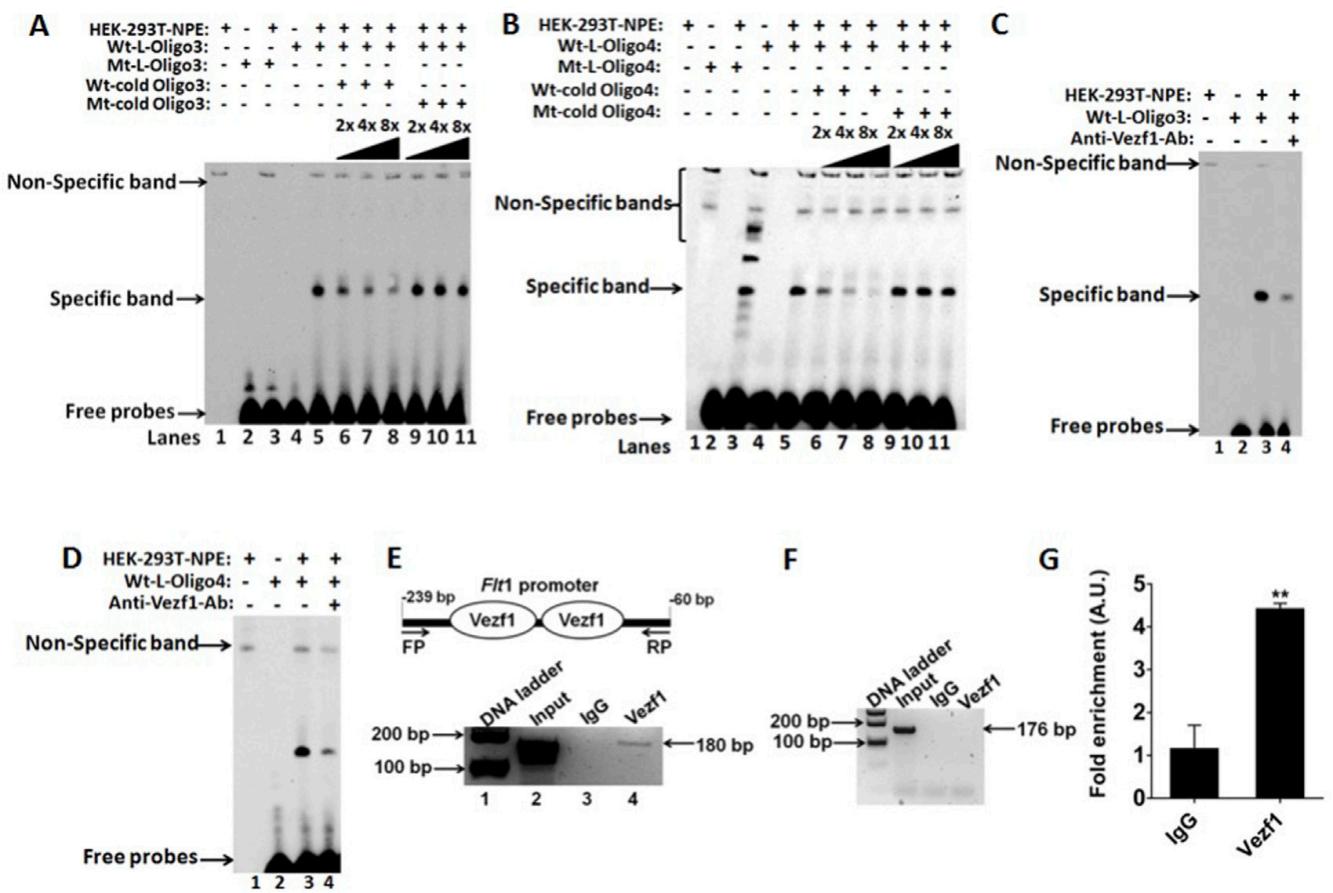
Electrophoretic mobility shift assays (EMSAs) and ChIP assay display binding of VEZF1 to *Flt1* promoter domains. **(A–B)** Specific shifts were observed in *Flt1* promoter oligo #3 and #4 when incubated with VEZF1 containing nuclear protein extracts (Lane five panels A and B). Competition EMSA performed with 2-, 4-, and 8-fold molar excesses of unlabeled (cold) wild-type oligos (lanes 6–8) resulted in a decrease of this shift whereas molar excess of cold mutant oligos (mutated for the VEZF1 binding sites) had no impact on this shift (lane 9–11). **(C–D)** Antibody supershift/interference assay for VEZF1: Addition of a VEZF1 antibody in the assay resulted in a high reduction of the shift with both oligo #3 and #4 confirming the shift to be resulting from VEZF1 protein in the nuclear extract (Lane 4, panels C, D). wt, wild-type; Mut, mutant. **(E–F)** PCR amplification of VEZF1 binding region in *Flt1* promoter using DNA obtained from a VEZF1 chromatin pull down (ChIP) of D4 embryoid bodies. PCR amplification of a non-specific Gapdh promoter region showed no enrichment validating the VEZF1 specific binding. IgG was used as a negative control for the ChIP studies. **(G)** Quantitative PCR shows an over 4-fold enrichment of VEZF1 binding to the *Flt1* promoter over the IgG.

Because the 0.7 kb *Flt1* promoter domain governing the *Flt1* gene transcription contained several potential binding sites for VEZF1 (Figure 2A), we tested the binding capacity of VEZF1 with synthetic double-stranded oligonucleotides (wild type/mutant) (Table 1) representing the *Flt1* promoter segments using EMSAs. The biotin-labeled *Flt1* oligonucleotide (Wt-L-Oligo3) yielded the specific protein/DNA complex (Figure 3A, lane 5) with HEK293 nuclear protein extracts, whereas the labeled mutant oligo did not yield any specific complex (Figure 3A, lane 3). Intensity of this shift was reduced in a dose dependent manner with addition of molar excesses of unlabeled wild-type oligo (Figure 3A, lanes 6–8), whereas the unlabeled mutated oligo did not show any competition effect (Figure 3A, lanes 9–11). Similarly, an EMSA with another *Flt1* oligonucleotide (Wt-L-Oligo4) displayed formation of one specific complex (Figure 3B, lane 5) that was inhibited by unlabeled wild type oligonucleotide in a dose dependent manner (Figure 3B, lanes 6–8); the intensity of this specific band was not changed in the presence of unlabeled mutant oligonucleotide (Figure 3B, lanes 9–11). Incubation with VEZF1 antibody decreased the intensity of the specific bands using either Wt-L-Oligo3 or Wt-L-Oligo4 (Figures 3C, D), lane 4), respectively, suggesting the specificity of VEZF1 binding to the with *Flt1* promoter oligoes.

We next validated VEZF1 binding to the endogenous *Flt1* promoter *in vivo* by pursuing ChIP assays. Formaldehyde-cross-linked, sonicated chromatin from day 4 EBs (derived from iHA-Etv2 mESCs) were immunoprecipitated using a VEZF1 antibody. PCR amplification resulted in the detection of *Flt1* promoter specific product from the VEZF1 pull-down (Figure 3E, lane 4), whereas the IgG control ChIP had no enrichment (Figure 3E, lane 3). PCR amplification of the *Gapdh* promoter region having no putative VEZF1 binding sites demonstrated the specificity of the VEZF1 antibody (Figure 3F) and the ChIP assay. Quantitative PCR analysis revealed a 4.0-fold enrichment of VEZF1 binding to the *Flt1* promoter region over the IgG control (Figure 3G). These results established that VEZF1 displayed robust interaction with the chromatin segments harboring the *Flt1* promoter domain.

### Absence of VEZF1 impairs hematoendothelial development in EBs

**FIGURE 4:**
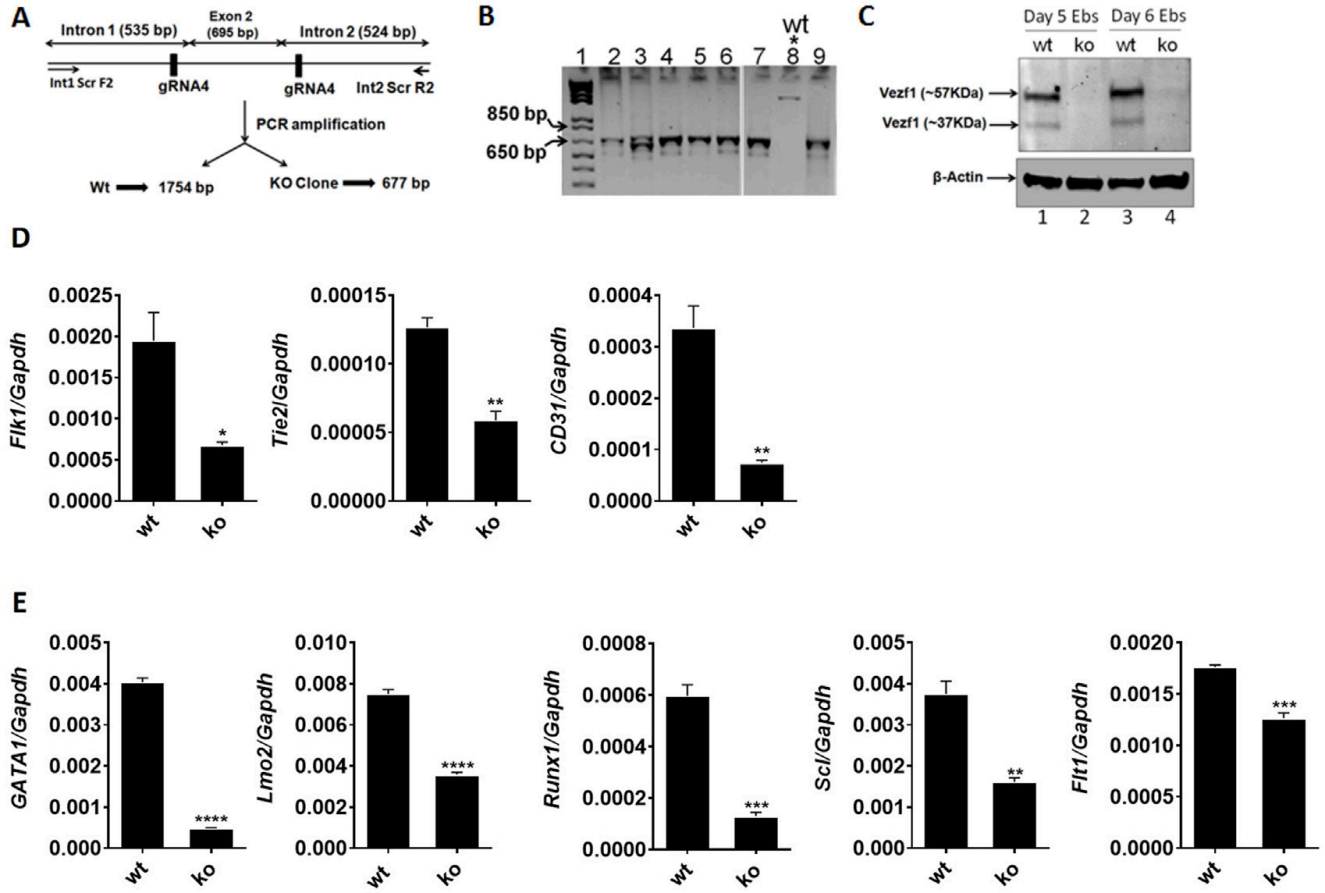
Generation and functional characterization of *Vezf1* knock out ES cells. **(A)** Schematic representation of the *Vezf1* deletion in mouse embryonic stem cells. Exon two of *Vezf1* was deleted with two guide RNAs flanking it. Deletion of the exon two gives rise to a deletion product of 677 bp with a primer pair binding in intron 1 and two of the *Vezf1* gene, whereas the WT locus would give a 1,754 bp product with the same primer pair. **(B)** PCR screening of *Vezf1* knock out ES cell clones. Clone nine was used as the *Vezf1* knock out line for further studies. **(C)** Absence of VEZF1 protein expression was verified in protein extracts from D4 and D6 embryoid bodies. **(D–E)** Gene expression analysis of D4 and D6 embryoid bodies showed significant downregulation of hematoendothelial marker genes in the absence of VEZF1. wt = wild type, ko = knockout. *p < 0.05, **p < 0.01, ***p < 0.001, and ****p < 0.0001.

The ES/EB system recapitulates the mechanisms that govern embryonic development (Keller, 1995). To establish the functional role of VEZF1 in hematoendothelial cell lineage development and differentiation, we generated *Vezf1* ^−/−^ mouse ES cells (ko) using CRISPR/Cas9 technology (Figure 4A) and deleted exon 2. The ES cell clone with homozygous deletion of the exon two was verified by PCR (Figure 4B). We used the ES/EB differentiation system to further characterize the *Vezf1* ^−/−^ ES cells. Western blot analysis of protein lysates from D5 and D6 EBs confirmed the absence of VEZF1 protein in the ko cells (Figure 4C). To assess whether the loss of VEZF1 leads to changes in expression levels of endothelial/hematopoetic markers, qPCR analysis was performed with total RNA isolated from D4, D6 EBs (wt and ko). The hematoendothelial markers (*Flk1*, *Tie2* and *CD31)* were significantly downregulated in the *Vezf1* ^−/−^ D4 EBs (Figure 4D), whereas early hematopoiesis markers *Gata1*, *Lmo2*, *Runx1*, *Scl*, and *Flt1* were downregulated in *Vezf1* ^−/−^ D6 EBs compared to the WT control EBs (Figure 4E).

### Overexpression of VEZF1 in embryonic stem cells enhances hematoendothelial potential during ES/EB differentiation

**FIGURE 5:**
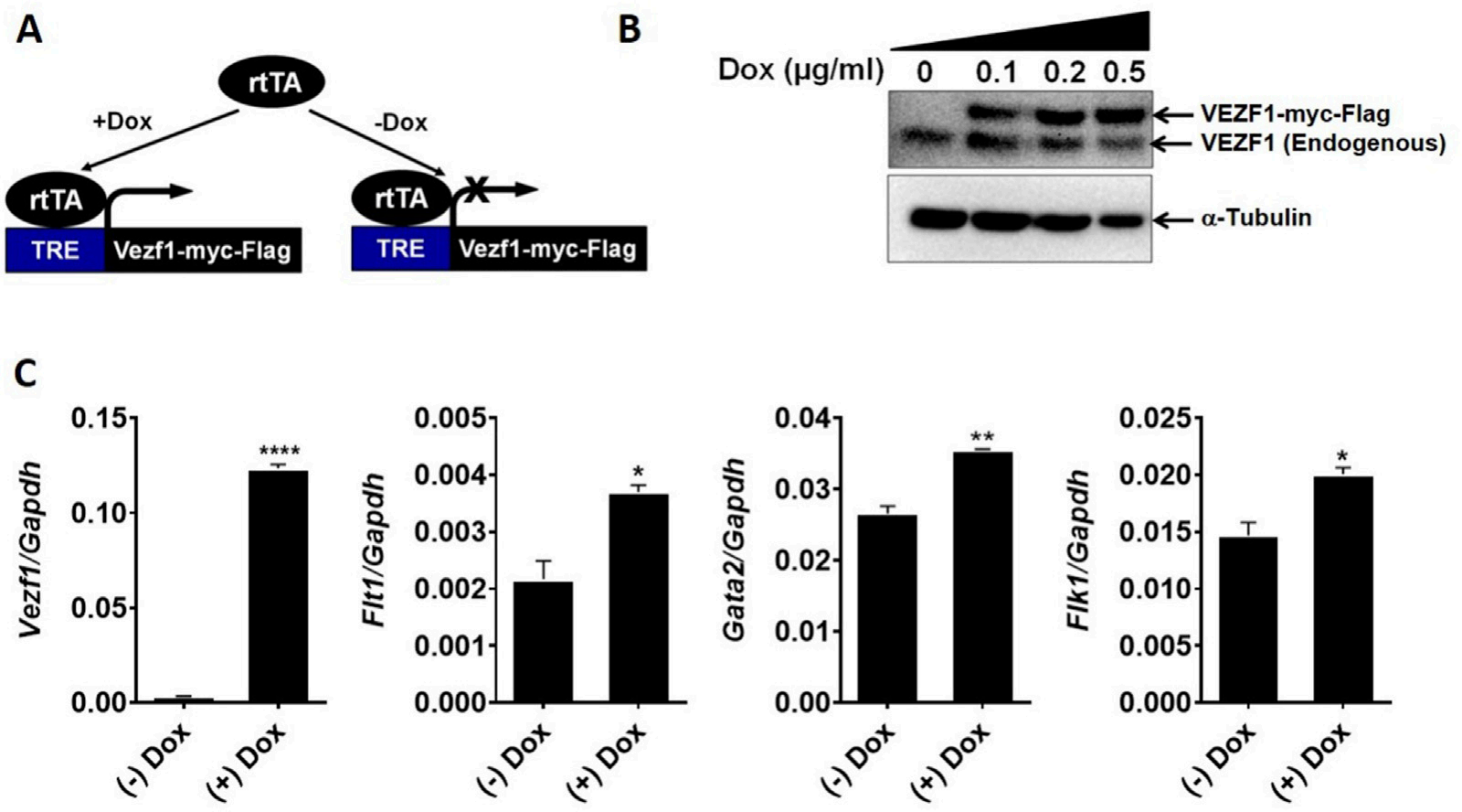
Generation and functional characterization of *Vezf1* overexpression ES cells. **(A)** Schematic of the Dox-inducible Vezf1 overexpression ES cells. **(B)** Overexpression of VEZF1 was verified by western blotting following treatment with different dosage of Dox. Tubulin was used as a loading control. **(C)** Overexpression of VEZF1 induced expression of hemato-endothelial marker genes in D4 EBs. *p < 0.05, **p < 0.01, ***p < 0.001, and ****p < 0.0001.

Our data showed that lack of VEZF1 diminished the expression of hematopoietic and endothelial markers in the differentiating ES/EB system. To further examine these results, we engineered an ES cell line in which VEZF1 could be induced by doxycycline using a cassette exchange recombination strategy (Iacovino et al., 2014) (Figure 5A). Induction of VEZF1 expression was confirmed by western blot and qPCR techniques, respectively (Figures 5B, C). The expression of VEZF1 was induced in differentiating EBs from D2 to D4. Gene expression analysis of hematoendothelial markers using RNA from D4 EBs showed the upregulation of *Flt1*, *Gata2*, and *Flk1* transcripts following VEZF1 overexpression compared to the control EBs, which further established the role of VEZF1 in hematoendothelial progenitor cells.

## Discussion

The first differentiated cell types to appear during vertebrate embryonic development are the hematoendothelial lineages. Vascular development involves two distinct stages, vasculogenesis and angiogenesis (Poole and Coffin, 1989; Marcelo et al., 2013; Park et al., 2013; Koyano-Nakagawa and Garry, 2017). Initially during vasculogenesis, the mesoderm-derived angioblasts migrate from the primitive streak and coalesce to form a vascular plexus (Coffin et al., 1991; Tanaka et al., 2014; Eliades et al., 2016). Subsequent sprouting/splitting, branching and differentiation and stabilization of the vascular plexus give rise to the mature blood vasculature which is termed angiogenesis (McGrath et al., 2003). These complex developmental processes require defined spatial and temporal expression and co-ordination of a large number of genes. The Ets family of transcription factors play functional roles during embryonic development including haematopoiesis and vasculogenesis (Sharrocks, 2001; Meadows et al., 2011). Among the ETS factors, the pioneer factor, ETV2 (Gong et al., 2022), plays a non-redundant role particularly during embryonic hematopoiesis and vasculogenesis as the absence of ETV2 expression leads to a complete absence of hematoendothelial lineages in mouse (Lee et al., 2008; Ferdous et al., 2009) and pig mutants (Das et al., 2020). Moreover, overexpression of ETV2 alone can reprogram non-endothelial cells to the endothelial lineage (Gong et al., 2022). To harness the therapeutic possibilities of ETV2 in regenerative medicine, an enhanced understanding of the regulatory mechanisms, molecular functions, networks and interacting partners that facilitate its activity is essential.

The zinc finger transcription factor *Vezf1* was discovered as a gene specifically expressed in the endothelial precursor cells during embryonic development (Xiong et al., 1999). *Vezf1* knockout mice resulted in embryonic lethality due to vascular remodeling defects, but did not impact other lineages (smooth muscle and metabolic pathways). *In vitro* studies utilizing *Vezf1* ^*−/−*^ embryoid bodies have been reported to have inefficient differentiation into endothelial cells, improper vascular network with dramatic vascular sprouting defects as well as defects in hematopoietic cell lineages (Zou et al., 2010). Both *in vitro* and *in vivo* analyzes have established a vital role for *Vezf1* in multiple processes of vascular development (Miyashita et al., 2004; Kuhnert et al., 2005; Miyashita and Sato, 2005; Zou et al., 2010; Bruderer et al., 2013; AlAbdi et al., 2018). However, the interacting partners, target genes and mechanisms of *Vezf1* involvement in this process is not fully understood.

A variety of transcription factor classes including ETS, Forkhead, GATA, Kruppel-like and SOX family of transcription factors have been shown to be involved in distinct stages of vascular development from mesodermal progenitors to differentiated endothelium (Zape and Zovein, 2011). ETV2, specifically, has been shown to form regulatory circuits with other transcription factors like FLI1, GATA2, TAL1, and OVOL2 to control the early hemangioblast and subsequent hematopoietic and endothelial development (Kataoka et al., 2011; Kim et al., 2014; Shi et al., 2014). We have previously reported the direct physical interaction between ETV2 and GATA2 to form a complex that regulates the development of both endothelial and hematopoietic lineages *via* a common downstream target gene *Spi1* (Shi et al., 2014). Furthermore, ETV2 has been shown to collaborate with FOXC2 to activate a subpopulation of the endothelial molecular program (De Val et al., 2008; Robinson et al., 2014). In the current study, we have identified VEZF1 as a direct binding partner and cofactor for ETV2 using an array of biochemical techniques. This interaction augmented the functional role of ETV2 during hematoendothelial development. Furthermore, our studies revealed that *Flt1* is a common downstream target gene of ETV2 and VEZF1.

Other than the binding partners and cofactors associated with ETV2 in regulating the hematoendothelial development, we have recently demonstrated the epigenetic regulatory mechanisms of ETV2 whereby it functions as a pioneer factor to bind nucleosomal DNA independently and relaxes closed chromatin around downstream factors to regulate endothelial development (Gong et al., 2022). ETV2 has been shown to recruit the ATP-dependent chromatin remodeler, BRG1 (SMARCA4), to sustain an open configuration and increase H3K27ac deposition near the downstream endothelial genes. VEZF1 also has been shown to be involved in epigenetic regulation through widespread binding at CpGi’s present in the promoters, enhancers and insulator regions of genes (Gowher et al., 2008; Gowher et al., 2012). So, the combinatorial role played by ETV2 and VEZF1 together in achieving epigenetic modifications of surrounding the endothelial development related genes require further exploration. These studies also emphasize the importance of interacting factors and their ability to mediate context dependent functional roles for ETV2.

In summary, our studies discovered VEZF1 as a novel binding partner for ETV2. These two factors were shown to be coexpressed in the endothelial lineage and coordinately activate gene expression. These studies further establish important mechanisms that govern hematoendothelial lineage development. Future studies will be needed to further explore the role of ETV2 and VEZF1 to commonly regulate the chromatin landscape during embryogenesis.

## Dedication

These studies and the manuscript are dedicated to VG who prematurely passed away on 2 May 2021.

## Data Availability

The original contributions presented in the study are included in the article/Supplementary Material, further inquiries can be directed to the corresponding authors.

## Abbreviations

ChIP, chromatin immunoprecipitation; EMSA, electrophoretic mobility shift assay; HEK, human embryonic kidney; HA, Hemagglutinin; E, embryonic day; Wt, wild-type; Mut, mutant; gRNA, guide RNA; EB, embryoid body; mES cells, mouse embryonic stem cells; qPCR, quantitative PCR; HET, heterozygous; D, day; GST, Glutathione S-Transferase; CoIP, co-immunoprecipitation; Dox, Doxycline; MEF, mouse embryonic fibroblast.
