# Supplementary material for: ETV2 and VEZF1 interaction and regulation of the hematoendothelial lineage during embryogenesis
Source: Front Cell Dev Biol. 2023 Feb 27;11:1109648. doi: 10.3389/fcell.2023.1109648 (PMC10009235; doi:10.3389/fcell.2023.1109648)
Supplement: Supplementary file 1 [file Image1.pdf]

Fig.S1

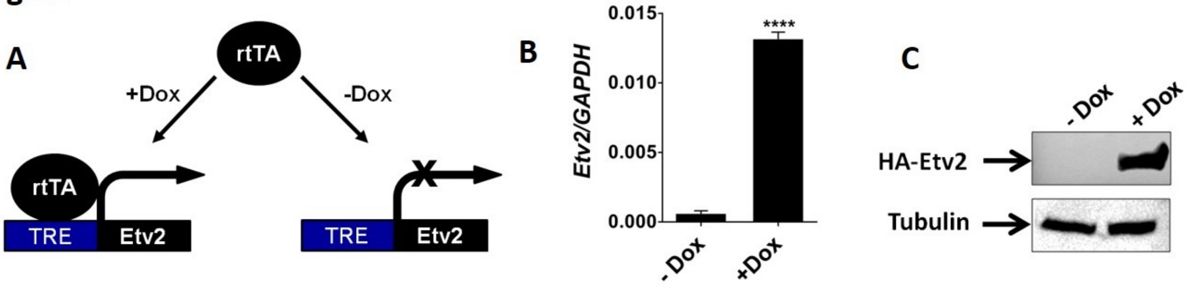

Fig.S1 iHA-Etv2 Embryonic stem cells. (A) Schematic of the Dox-inducible iHA-Etv2 embryonic stem cells. (B) Induction of Etv2 following Dox-treatment is verified by both quantitative RT-PCR and western blotting (C). ****p<0.0001.

Fig.S2

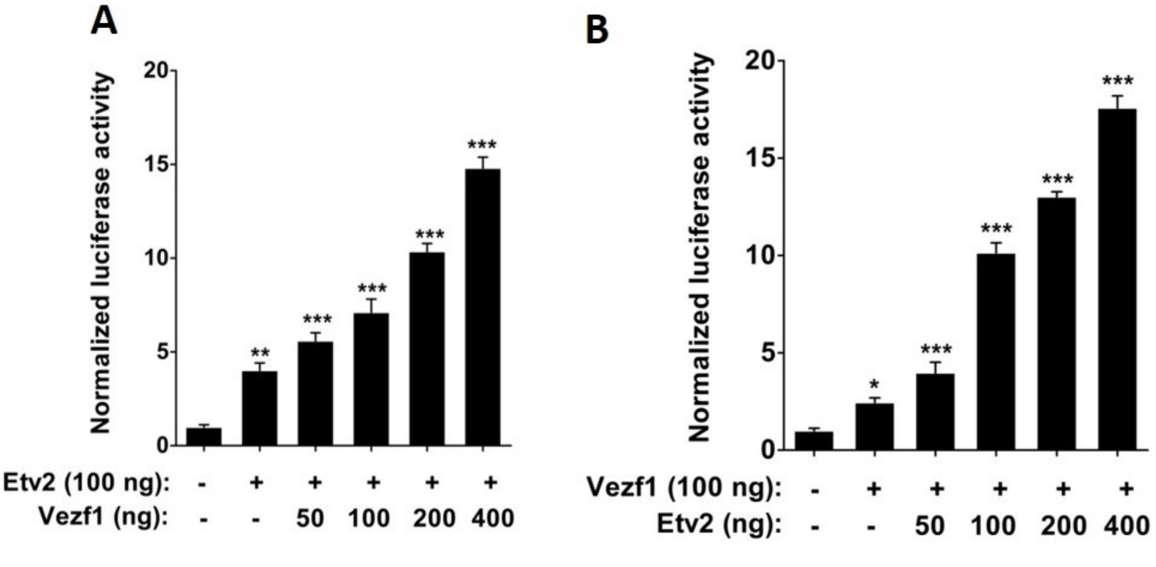

Fig.S2 Transactivation of *Flt1* promoter by ETV2 and VEZF1 in a dose-dependent manner. VEZF1 (A) and Etv2 (B) activate the Flt1 promoter driven luciferase activity in a dose-dependent manner when co-transfected with a constant amount of the other. *p<0.05, **p<0.01, ***p<0.001 and ****p<0.0001.
